# Identifying the sequence specificities of circRNA-binding proteins based on a capsule network architecture

**DOI:** 10.1186/s12859-020-03942-3

**Published:** 2021-01-07

**Authors:** Zhengfeng Wang, Xiujuan Lei

**Affiliations:** 1 School of Computer Science, Shaanxi Normal University, Xi’an, 710119 China; 2 College of Information Science and Engineering, Guilin University of Technology, Guilin, 541004 China

**Keywords:** Circular RNA, RNA-binding protein, Sequence specificities, Capsule network

## Abstract

**Background:**

Circular RNAs (circRNAs) are widely expressed in cells and tissues and are involved in biological processes and human diseases. Recent studies have demonstrated that circRNAs can interact with RNA-binding proteins (RBPs), which is considered an important aspect for investigating the function of circRNAs.

**Results:**

In this study, we design a slight variant of the capsule network, called circRB, to identify the sequence specificities of circRNAs binding to RBPs. In this model, the sequence features of circRNAs are extracted by convolution operations, and then, two dynamic routing algorithms in a capsule network are employed to discriminate between different binding sites by analysing the convolution features of binding sites. The experimental results show that the circRB method outperforms the existing computational methods. Afterwards, the trained models are applied to detect the sequence motifs on the seven circRNA-RBP bound sequence datasets and matched to known human RNA motifs. Some motifs on circular RNAs overlap with those on linear RNAs. Finally, we also predict binding sites on the reported full-length sequences of circRNAs interacting with RBPs, attempting to assist current studies. We hope that our model will contribute to better understanding the mechanisms of the interactions between RBPs and circRNAs.

**Conclusion:**

In view of the poor studies about the sequence specificities of circRNA-binding proteins, we designed a classification framework called circRB based on the capsule network. The results show that the circRB method is an effective method, and it achieves higher prediction accuracy than other methods.

## Background

Circular RNAs (circRNAs) are a category of noncoding RNAs with covalent closed structures and no polyadenylated tails [1]. These RNAs are formed by a back-splicing process in which the downstream 5′ splice donor is reverse-spliced to the upstream splice acceptor, a process regulated by *cis* elements and *trans* protein factors [2]. For a long time, circRNAs were thought to be splicing errors expressed at low levels [3]; now, benefitting from the advent of high-throughput sequencing experimental technology, they have been demonstrated to be a class of abundant, stable and conserved RNAs across species [4]. Some circRNAs have tissue-specific, cell-specific expression patterns [5] and participate in various human disorders [6–8]. Still, little is known about the formation and function of circRNAs, while recent studies have shown that circRNAs could serve as “sponges” of miRNAs, playing key roles in the posttranscriptional regulation of RNAs [9–11]. Increasingly, studies have revealed that some circRNAs may “sponge” RBPs (RNA-binding proteins) [12–15], thereby modulating protein–protein interactions.

RBPs are a class of proteins that can interact with RNA molecules and are associated with the metabolic processing of RNAs. Recent studies have shown that RBPs are involved in almost all phases of the circRNA lifecycle [16]. Zhang et al. [17] found that overexpression of QKI-5 notably increased circ-MTO1 (hsa_circ_0007874) expression in lung adenocarcinoma, suggesting that QKI-5 promotes the production of circ-MTO1. Wang et al. [18] found that eukaryotic initiation factor 4A3 (EIF4A3) induced circMMP9 (hsa_circ_0001162) cyclization and increased circMMP9 expression in glioblastoma multiforme (GBM). He et al. [19] demonstrated that FUS binds to and promotes the production of hsa_circ_0000005 to regulate glioma angiogenesis. Moreover, the binding of circRNAs and RBPs may have bidirectional effects, and circRNAs could act as dynamic scaffolding molecules that modulate proteins. For example, Du et al. [20] showed that ectopic circ-Dnmt1 (hsa_circRNA_102439) could bind to AUF1 and promote AUF1 nuclear translocation. In addition, there are research reports that MOV10 binding circ-DICER1 (hsa_circ_0033079) regulates the cell viability, migration, and tube formation of glioma-exposed endothelial cells (GECs) [21]. Hong et al. [22] inferred that circFNDC3B (hsa_circ_0006156) promoted CD44 expression via IGF2BP3 and that IGF2BP3 could affect the function of circFNDC3B to a certain extent. Due to the tertiary structure of circRNAs, the protein-binding capacity of circRNAs is likely to be greater and more complex than that of linear RNAs [23], and RBPs bound to circRNAs are not replaced by ribosomes. Therefore, to understand the formation and function of circRNAs, it is essential to study the interaction mechanism between circRNAs and RBPs.

To date, these interactions have been analysed mainly through biological experimental methods, such as RNA immunoprecipitation (RIP) [24] or RNA pull-down assays [25]. In the RNA pull-down assay, the probe pulls down the RNA to analyse the associated proteins. A protein is immunoprecipitated to analyse associated RNA in the RIP assay. Recently, CLIP-seq [26] has become a useful experimental strategy that can detect potential binding sites on unreported sequences. CLIP-seq contains several variants, including HITS-CLIP [26], PAR-CLIP [27], and iCLIP [28]. Benefiting from these high-throughput biological experiments, several databases of circRNAs have been built to study the interactions between circRNAs and RBPs. For example, circBase collects and unifies datasets of circRNAs and provides scripts to identify circRNAs in sequencing data [29]. The RBP binding sites, miRNA target sites and ORFs (potential open reading frames) on cancer-specific circRNAs are provided in the CSCD database [30]. CircRic [31] analysed the association between circRNAs and proteins in 935 cancer cell lines across 22 cancer lineages from Cancer Cell Line Encyclopedia (CCLE). starBase [32] is mainly focused on miRNA-target and RBP-target interactions. CircInteractome [33] provides potential binding sites on junctions and junctions flanking RBPs and miRNAs within circRNAs.

Several significant discoveries have been made through these biological experimental technologies; however, they are expensive, labour-intensive and time-consuming. In contrast, high-throughput biological experimental methods could provide a large number of available data sources for computation-based methods [34, 35]. For example, Alipanahi et al. [36] proposed a classification method to identify the RNA-binding sites in proteins based on RNA high-throughput sequencing data. Recently, based on circRNA biological experimental data, a computational framework was constructed by employing positive unlabelled learning (P-U learning) to predict unknown circRNA-RBP interaction pairs with the kernel model MFNN (matrix factorization with neural networks) in our previous work [37]. CRIP [38] and CSCRSites [39] employed different deep learning frameworks to identify the binding sites within circRNAs. CircSLNN [40] treats the prediction task of RBP binding sites as a sequence labelling problem to identify RBP binding sites on circRNAs. CRIP and CSCRsites accept a fixed-length circRNA segment, and noisy nucleotides may be generated that affect the outcome of the prediction. CircSLNN avoids the problem of fixed-length binding sites, but it also provides a new problem of sample imbalance.

**Fig. 1:**
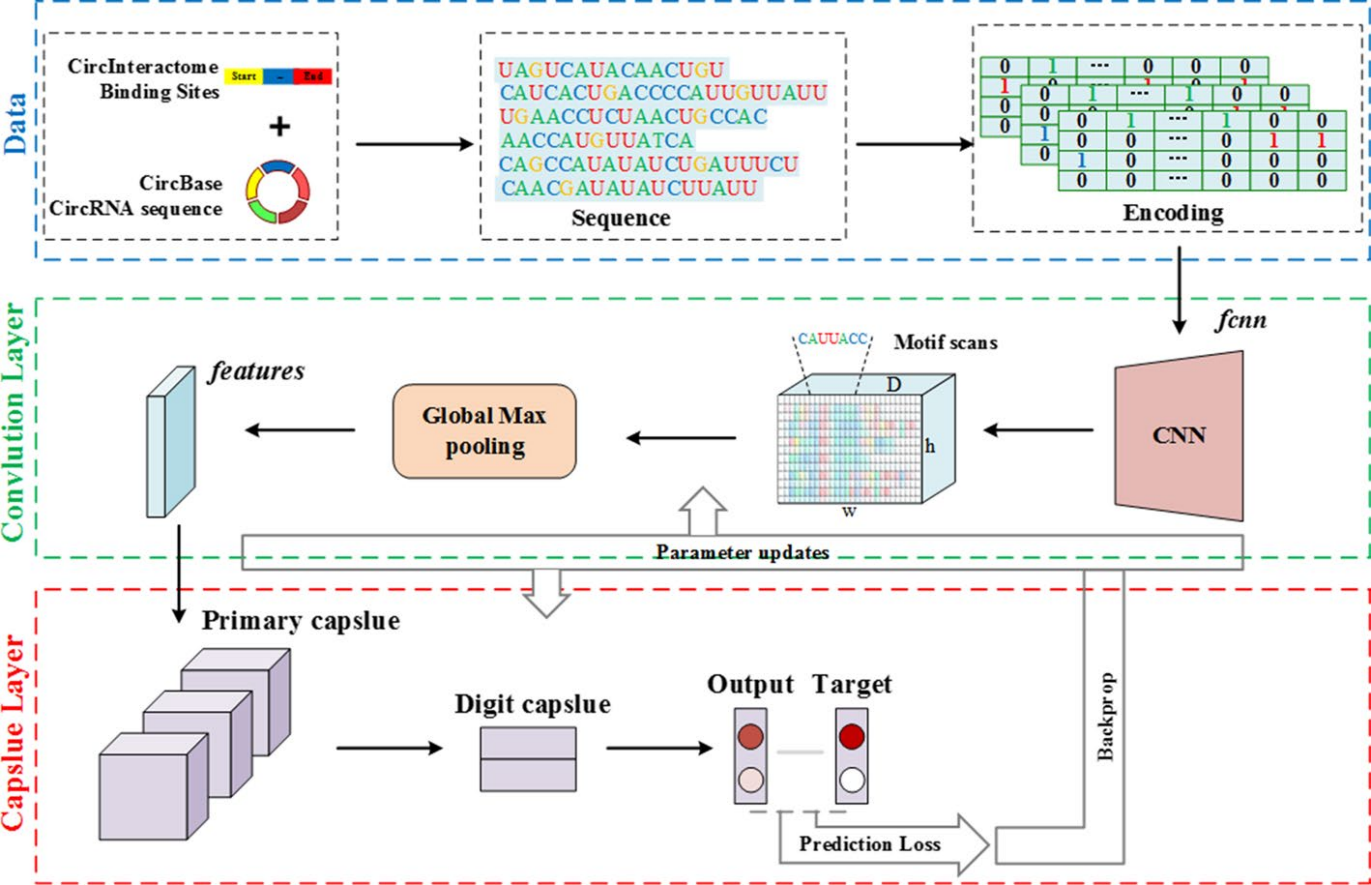
Schematic diagram of circRB model construction. The sequence features of circRNAs are extracted by convolution operation, and then two dynamic routing algorithms in the capsule network are employed to discriminate between different binding sites by analysing the convolution features of binding sites

In this study, we design a prediction model named circRB (Fig. 1) to identify the sequence specificities of circRNA-binding proteins. The model allows for various lengths of circRNA fragments as input. The convolution operation is employed to extract the original sequence features of circRNA fragments. The sequence features are fed to a capsule network, discriminating the binding sites on circRNAs. We test circRB on seven datasets and compared it with other existing methods. The experimental results show that our method is 0.03 on average higher than the other best methods regarding AUC. In addition, we compare the binding motif detected by this model to the existing RNA motif database, and some motifs on circular RNAs overlap with those on linear RNAs, especially in the QKI dataset. Finally, we apply this model to full-length circRNA sequences to predict binding sites and find potential binding sites with high scores in most known binding relationships. In conclusion, circRB is an effective prediction model for identifying RBP binding sites on circRNAs. We hope that our model will contribute to a better understanding of the mechanisms of the interactions between RBPs and circRNAs.

## Results

In this section, we first evaluate the performance of the circRB method. Then, circRB is compared with the existing deep learning-based methods for predicting RBP binding sites on the same dataset. Finally, we discuss the performance of circRB in the sequence specificities of circRNA-binding protein discovery.

### Training circRB and experimental settings

In the training phase, the optimization algorithm Adam is used to minimize the loss function. The batch size is set to 64. To accelerate operation and shorten the training time, the batch size can be modified to 512 on large datasets, such as EIF4A3. The models are trained and validated after each epoch until the losses are no longer reduced, which is selected as the best model. Generally, 30 epochs are sufficient, and we found that only 10–15 epochs are needed to obtain the optimal model in large datasets. In the training and testing phase, each dataset is divided into two groups with random sampling, namely, 20% for testing and 80% for training the model, and 5-fold cross validation is adopted to assess the model.

**Fig. 2:**
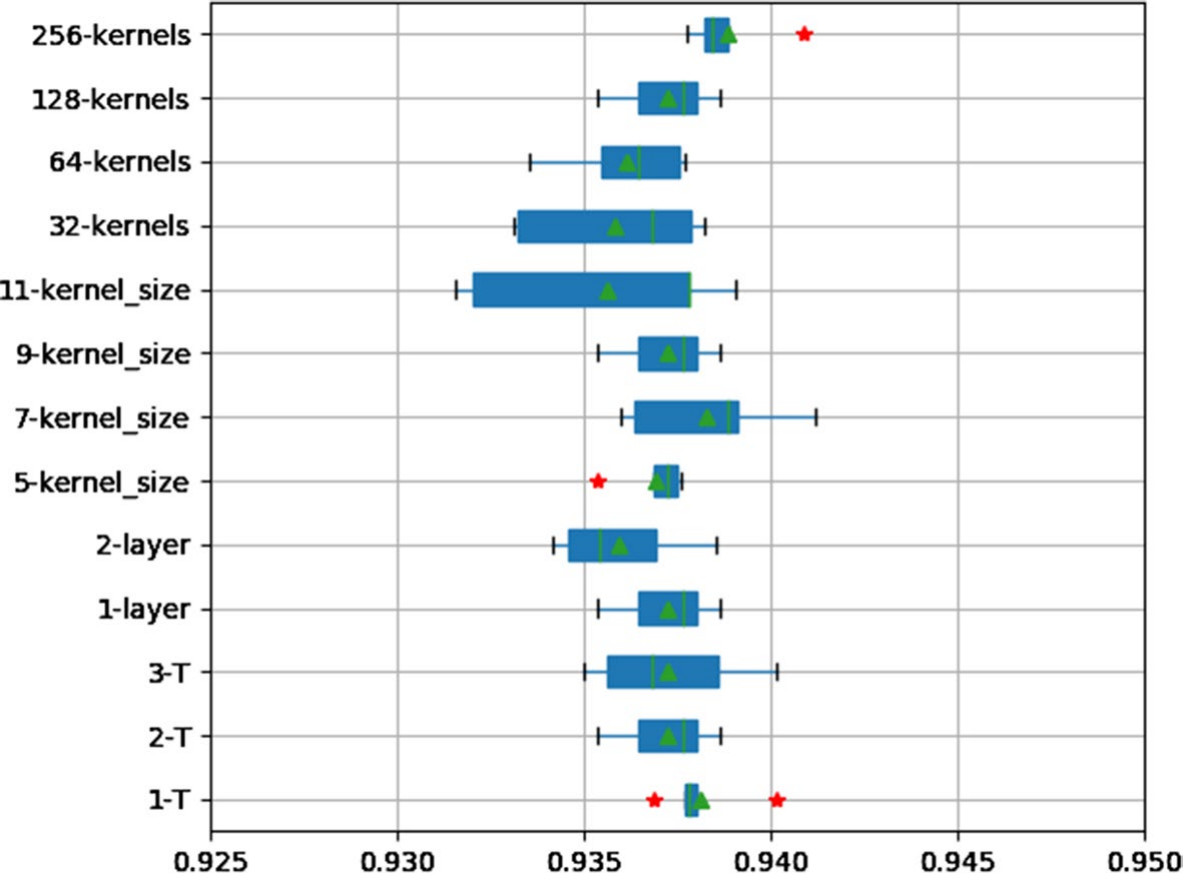
The distribution of AUCs across various parameters and structures

The parameters of a deep learning model often have a significant impact on its performance, such as the number of dynamic routing layers, the number of convolution layers, the number of convolution kernels, the size of the convolution window and other parameters in our model. We analysed the model parameters on the AUF1 dataset employing 5-fold cross validation. The results are shown in Fig. 2. The median values of AUCs convoluted by one convolution layer (1-layer, 0.9377) are higher than those of 2-layers (0.9354). Simultaneously, we also tested different convolution kernel window sizes, ranging from 5 to 11 (5…11-kernel_size). Figure 2 shows that both 7-kernel_size (0.9388) and 9-kernel_size (0.9377) achieve better results. The kernel size is set to 9, and the results of the model are relatively more stable. In addition, we also tested the effect of different numbers of convolution kernels on the performance of the model. When the number of convolution kernels reaches 128, the model obtains more ideal results (0.9377). Of note, the 256 convolution kernels are at risk of overfitting on some small data sets. Therefore, 256 convolution kernels are more suitable for datasets with a large amount of data. Overall, the model is insensitive to the parameters.

Finally, we adopted different numbers of kernels in the convolution layer for each dataset. It is usually set to 128 to achieve a better effect. If the data volume is large, it can be improved to 256. The kernel size of long sequence segments is 11, and that of shorter segments is 9. The activation function is ReLU (rectified linear unit) in the convolution layer. Sixteen or 32 capsules with 8-dimensional vectors are used in the primary capsule layer. Two capsules are constructed in the digital capsule layer.

### The effect of dynamic routing times

Generally, 2 dynamic routing cycles can achieve better performance in the capsule network framework, and more routing cycles may lead to worse results. We evaluated the effect of different dynamic routing times on the performance of the model on the AUF1 dataset employing 5-fold cross validation. The results are shown in Fig. 2. The 1-T, 2-T, and 3-T represent 1, 2, and 3 implementations of the dynamic routing algorithm, respectively. The AUC median values of 1-T and 2-T are 0.9378 and 0.9377, respectively. However, when the dynamic routing algorithm is executed twice, the results of the model are relatively more stable. This finding indicates that the generalization ability of the model is better when the dynamic routing algorithm is executed twice. In addition, the performance of the model decreases (0.9368) when the dynamic routing algorithm is executed three times. Simultaneously, the performance of the model is insensitive to dynamic routing times T. Finally, we set the dynamic routing times T as 2.

### Max pooling improves the prediction performance

**Fig. 3:**
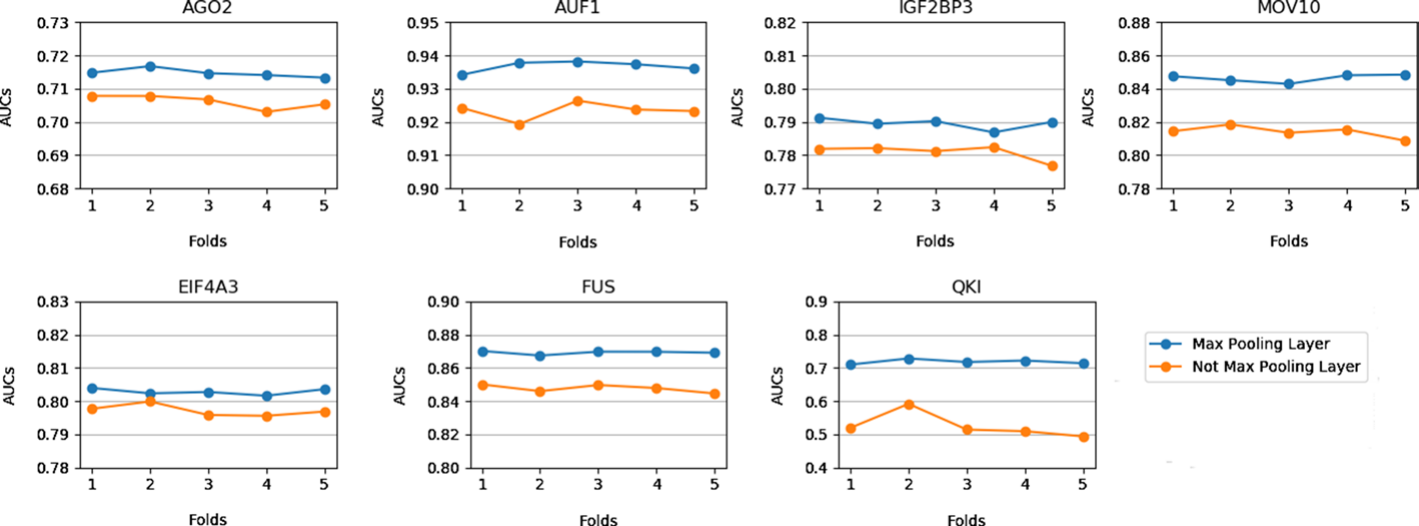
The max pooling improves the prediction performance. The blue line represents the result with the max pooling layer, and the orange line represents the result without the max pooling layer

In the standard capsule network proposed by Sabour et al. [41], for the pooling layer to be deleted, some feature information may be lost due to the pooling operation. However, the pooling layer can significantly improve the prediction performance of the model, as shown in Fig. 3.

On the seven datasets, the model with the pooling layer obtained the highest AUC values, and the AUC value of each fold fluctuated slightly. In addition, the pooling operation can also greatly save computing hardware resources and speed up the calculation. Therefore, the pooling layer was still adopted in this study.

### Performance evaluation of circRB

**Fig. 4:**
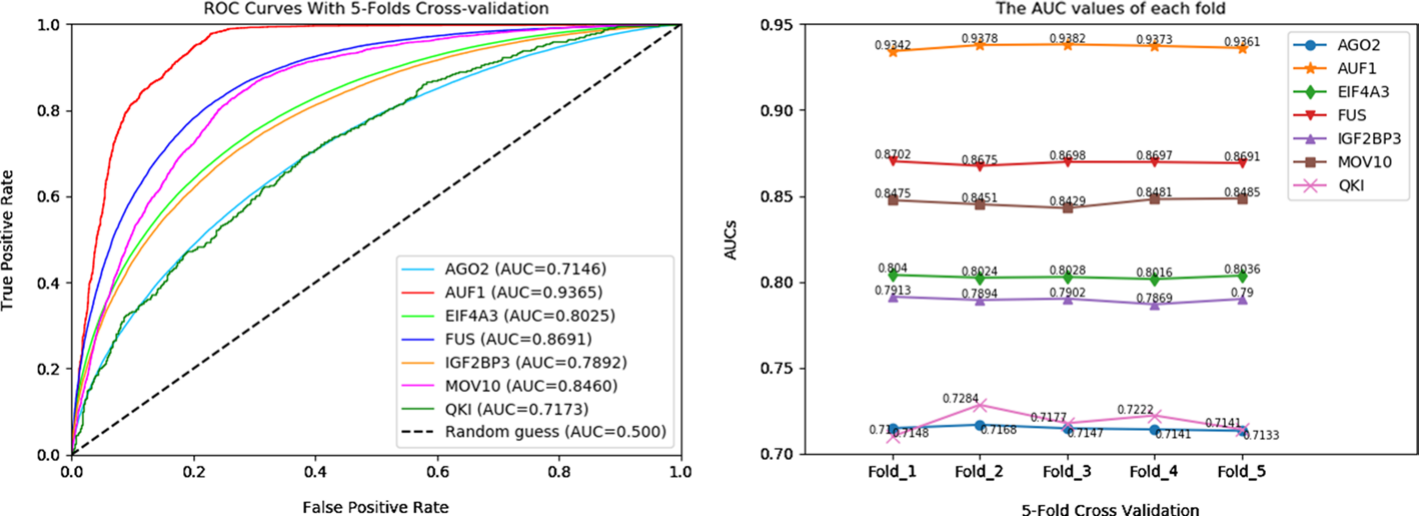
The ROC curves obtained and the AUC values of each fold for circRB with 5-fold changes in seven RBP datasets

In this study, the area under the receiver operating characteristic curve (ROC_AUC) was used as a metric for model evaluation and comparison [42]. We performed experiments on seven RBPs datasets. For each dataset, 5-fold cross-validation was employed to evaluate the prediction model [43]. The training set was divided into two groups with random sampling (80% for training and 20% for testing). The ROC curves were obtained, and the AUC values of each fold for circRB with 5-fold cross validation on seven RBP datasets are shown in Fig. 4.

As shown in Fig. 4, circRB achieves the highest AUC values for most RBPs. The AUC values are higher than 0.8 on 4 out of the 7 datasets. The highest AUC value of 0.93 was obtained on the AUF1 dataset. However, the model obtained a lower AUC value on the QKI dataset. During the model training, we also found that more epochs are needed for model convergence on the QKI dataset. This fact may be caused by the small size of the QKI dataset. In addition, Fig. 4 shows that the AUC value of our model varies little in each fold, with an amplitude of 0.005, indicating the robustness of our model. These results indicate that circRB is an effective model for predicting circRNA-binding sites.

### Comparing circRB with the existing deep learning methods

**Fig. 5:**
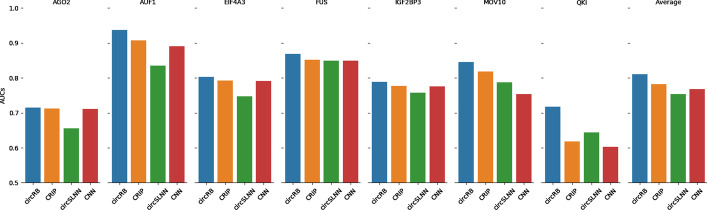
The AUC values obtained for each model with 5-fold cross validation

In recent years, several studies have analysed circRNA-binding sites using different methods. CRIP [38] predicts the RBP binding sites on circRNA by combining a convolution neural network (CNN) and a recurrent neural network (RNN). Different from CRIP, circSLNN [40] converts the prediction of binding sites on RNAs to a sequence labelling problem and classifies using a conditional random field (CRF) layer instead of a fully connected layer (FC). In this study, we compared our model with CRIP and circSLNN on seven RBP datasets with 5-fold cross validation. In addition, we also applied the convolution neural network applied on the same dataset. The results are shown in Fig. 5.

Figure 5 shows that the AUC values obtained by our model are all higher than those of the other existing methods (on seven datasets, the *P* values of circRB compared with other methods are all less than 0.05, as shown in Table 1). This is most evident on the QKI dataset. The average AUC value achieved by circRB (0.8107) on the seven RBP datasets was also significantly higher than that of CRIP (0.7824), circSLNN (0.7538) and CNN (0.7677). The AUC value is 0.7146, which is very close to CRIP (0.7120) and CNN (0.7116) on the AGO2 dataset. Of note, circSLNN achieves low AUC values on all datasets, which may be because circSLNN has obtained unbalanced positive and negative instances during model training after considering the problem of site prediction as a sequence labelling task. In addition, Fig. 5 shows that circRB performs significantly better than other methods on the QKI dataset. This finding validates the advantage of the capsule network in small-sample learning. The equivariance feature representation capacity makes the capsule network learn from a small-sample data, so it does not need as many samples as other neural networks [44].

**Table 1:** *P* values of circRB compared with other methods on the seven datasets

α = 0.05, *P* values	CRIP	circSLNN	CNN
circRB	0.032810	0.000319	0.020249

### Performance of circRB in motif discovery

In this section, the motifs learned by circRB on the positive instances of seven RBP datasets are aligned to the existing motifs using the web tool Tomtom with an *E* value $\le$ 0.05. Ray2013 Homo sapiens was selected as the desired motif database containing 102 RNA-binding motifs. We found that some motifs on circular RNAs overlap with those on linear RNAs, and the different RBPs have similar binding patterns on circular RNAs and linear RNAs.

**Fig. 6:**
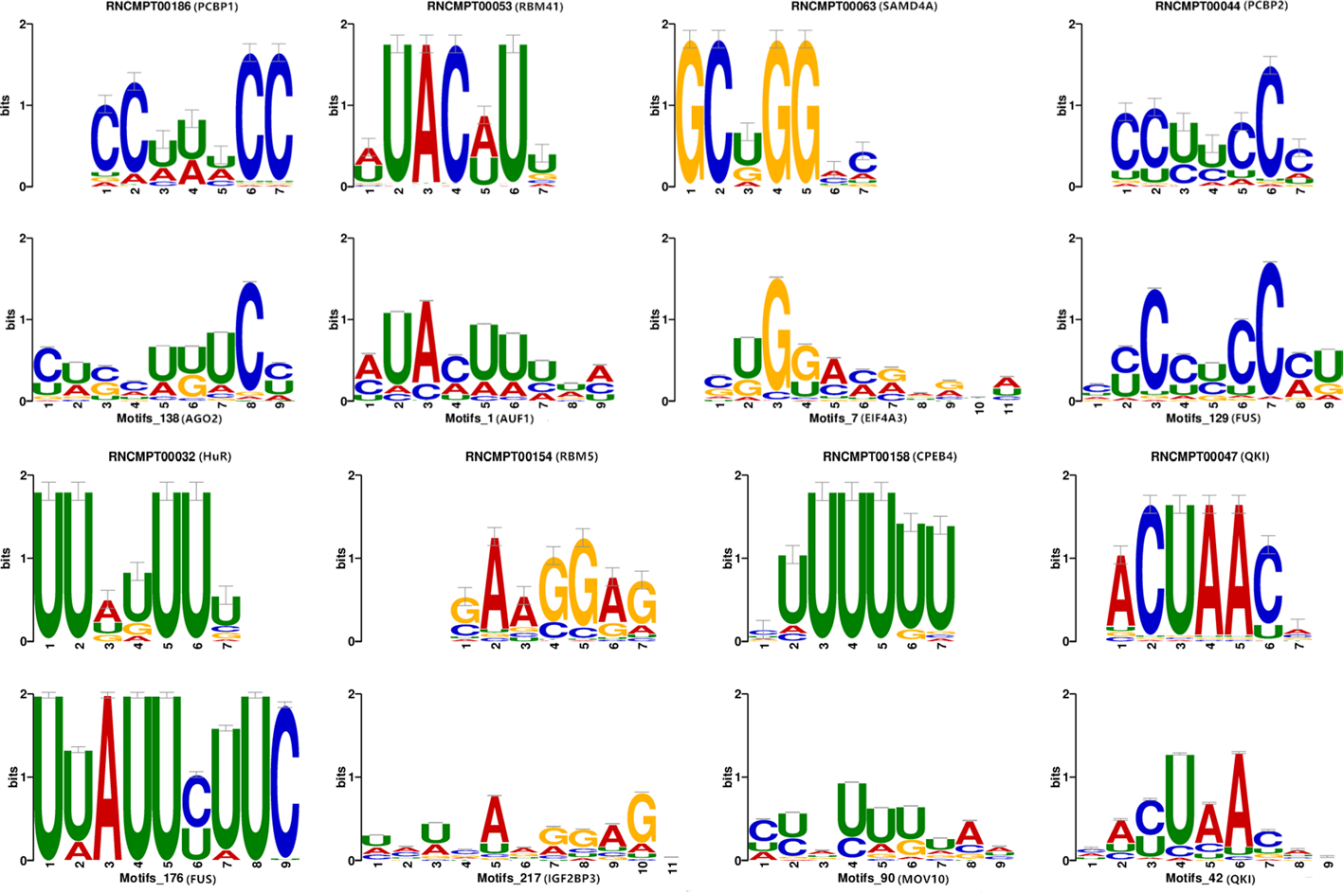
Sequence logos of matched motifs. For each plot, the motifs learned by circRB (bottom) are aligned with the known motif (top) from the Homo sapiens database by Tomtom

As shown in Fig. 6, the binding motif ‘ACUAAC’ is on the circRNA binding to QKI, and it also appears on linear RNA. Indeed, more than one-third of human circRNAs are strictly controlled by QKI and can promote the formation of circRNAs by binding to canonical motifs (ACUAACN_1___20_UAAC motif) on the flanking introns of circRNAs [17].

**Fig. 7:**
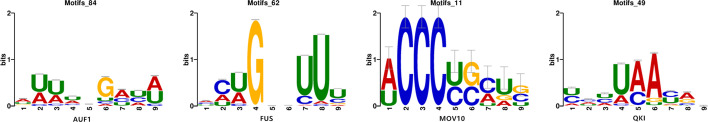
Motifs specifically found in the positive instances

The motifs learned by circRB contain relevant features to distinguish the positive and negative instances of RBP binding sites. Therefore, we also detected the motifs for the negative instances and compared them with those of positive instances with an *E* value $\le$ 0.001. Excitedly, we found that some motifs listed in Fig. 7 were only present in positive instances, although most motifs were also present in negative instances. As shown in Fig. 7, motif_49 of QKI contains the binding motif ‘UAAC’, which has been reported. Unfortunately, no significant positive motif was found in the other three RBP datasets. This is most likely due to the large size of the three datasets. Of the three datasets, IGF2BP3, which contains the lowest number of positive instances, also has more than 50,000 positive instances.

### Identification of RBP binding sites on full circRNAs

**Fig. 8:**
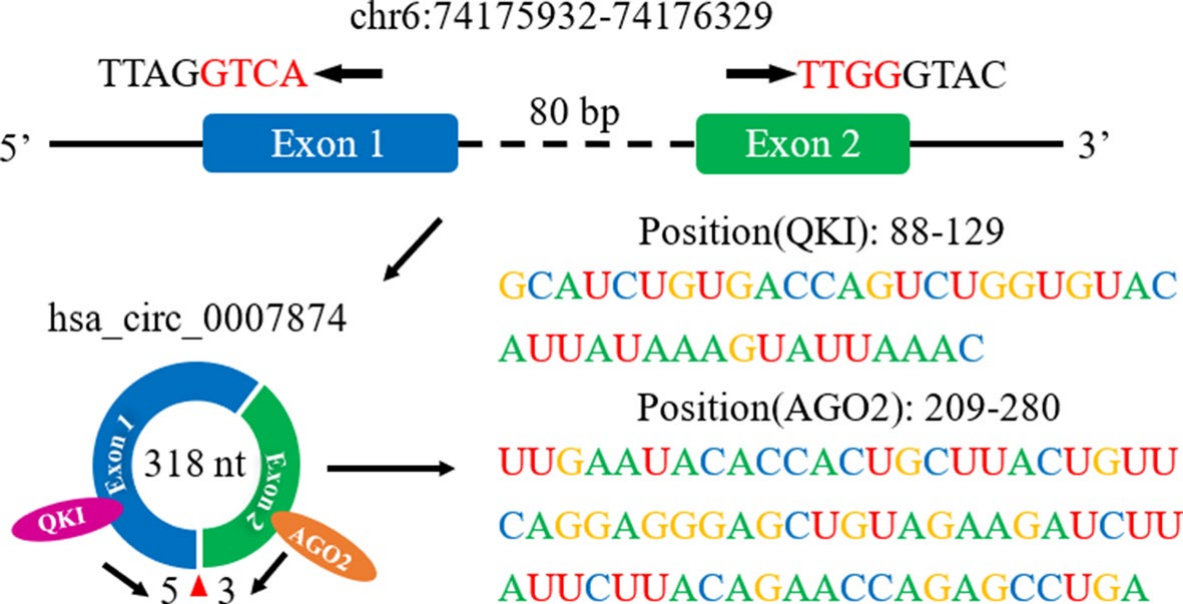
Binding positions of AGO2 and QKI on hsa_circ_0007874

An attempt was also made to assist current studies. We collected the reported full-length sequences of circRNAs combined with RBPs. These sequences are fed to the corresponding trained model. Finally, the possible binding positions and scores are obtained. We list the highest scoring fragments as possible binding sites for each circRNA sequence in Table 2. In Table 2, the first column is RBP, the second column is circRNA, the third column is the predicted potential binding site, and the fourth column is the probability that the location is a binding site. Except for AUF1(0.5640), most of the potential binding sites obtained high scores. This may be because all of the datasets used in the training of the model are from the standard circRNA sequences included in the circBase database, while hsa_circRNA_102439 is identified by the authors of the paper. Therefore, the features of this binding site are unprecedented in our model. Furthermore, because hsa_circ_0007874 could combine with QKI and AGO2, by further analysing the positions of binding sites on circRNA, we found that they are distributed on exon 1 and exon 2. As shown in Fig. 8, they are all close to the junction flanking.

**Table 2:** Sequence specificities binding RBPs on the reported full-length circRNA sequences

RBPs	circRNAs	Predicted positions	Binding scores
AGO2	hsa_circ_0001346	104–175	0.7812
	hsa_circ_0001946	458–529	0.8719
	hsa_circ_0006101	77–148	0.8536
	hsa_circ_0006117	173–244	0.8442
	hsa_circ_0007874	209–280	0.8182
AUF1	hsa_circRNA_102439	36–87	0.5640
EIF4A3	hsa_circ_0001162	70–235	0.6946
FUS	hsa_circ_0000005	41,833–41,888	0.8935
IGF2BP3	hsa_circ_0006156	330–471	0.8772
MOV10	hsa_circ_0033079	6120–6191	0.7526
QKI	hsa_circ_0007874	87–128	0.7430

## Discussion

Recent studies have demonstrated that circRNAs can interact with RNA-binding proteins (RBPs), which is also considered an important aspect for investigating the function of circRNAs. In this study, we design a capsule network-based model called circRB to identify the sequence specificities of circRNA-binding proteins. The sequence features of circRNA fragments are extracted through a convolution operation in the first layer of the circRB. The capsule network is employed to discriminate whether the fragments are the binding sites or not, by analysing the convolution features. circRB is trained and tested on the seven datasets, and it is also compared with other existing methods.

The experimental results show that the average AUC value of our model is 0.03 higher than other best methods. Furthermore, the binding motif detected by the circRB model is aligned to the existing RNA motif database, and we found that some motifs on circular RNAs overlap with that on linear RNAs, especially in the QKI data set. Finally, the circRB model was applied to the full-length circRNA sequences to predict binding sites, and excitingly, the potential binding sites with high scores were detected in most known binding relationships.

The circRB model has excellent performance and is comparable with other state-of-the-art methods on seven RBP datasets. The main highlights and the better performance of our model is mainly attributed to the following aspects: (1) the circRB model allows unequal circRNA fragments to be used as model inputs, and the learning bias caused by off-target nucleic acid sequence is avoided. (2) The capsule network could seize the characteristic that the binding direction of the binding sites is equivalent on circRNAs, thus improving the ability of site recognition. (3) The max pooling is still adopted, which improved the prediction performance of the circRB model. Despite the enhanced performance, circRB continues to underperform with data it has never seen before. In future research, we will collect more binding site information on circRNAs to improve the performance of circRB. We believe that circRB will make contributions to better understand regulatory functions of circRNAs.

## Conclusion

Because sequence specificities of circRNA-binding proteins are poorly studied, we designed a classification framework named circRB based on the capsule network. The results showed that circRB achieves higher prediction accuracy, and it is an effective classification method. In the future, we will attempt to build a web tool for binding site prediction, and we hope our model will contribute to better understanding mechanisms of the interactions between RBPs and circRNAs.

### Methods

#### Datasets and encoding

**Fig. 9:**
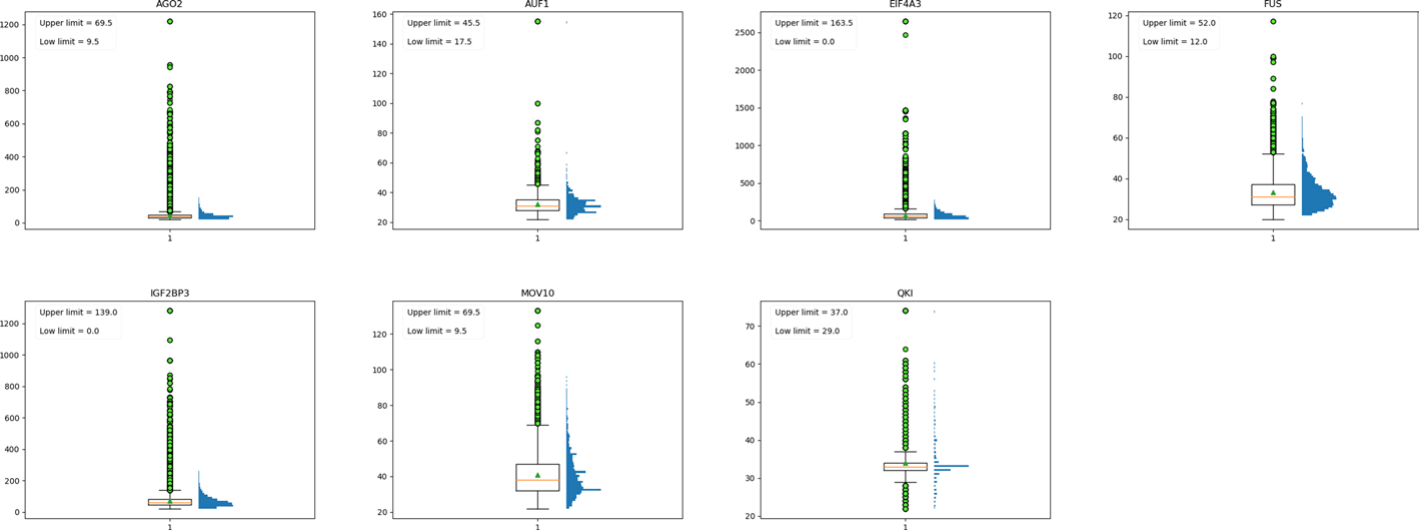
Statistical results of binding site length for seven RBPs

To identify the sequence specificities of circRNA-binding proteins, we constructed seven datasets of RBP-binding sites on circRNAs. These RBPs are involved in human disease processes by interacting with circRNAs, and they are included in the CircInteractome (circRNA interactome database) database. As shown in Table 3, information on the binding sites was extracted from the CircInteractome database. The spliced circRNA sequences were downloaded from the circBase database. There is overlap of binding sites in the CircInteractome database, especially highly overlapping sites. This fact may cause classification bias in the classification model. Hence, we removed the highly overlapping redundant binding sites. The negative instances were generated by dinucleotide-shuffling the binding site sequences. The bound sequences are shuffled in this way so that dinucleotide frequencies (AA, AC, …, GT, TT) from the original sequences are exactly preserved. For a classifier model, dinucleotide-shuffle could prevent the model from discriminating the foreground from the background depending only on the low-level statistics of genomic regions, such as CG dinucleotides [36]. This is a potential advantage over standard nucleotide shuffling. In general, parallel operation batch instances are used in deep learning models, and fixed-length sequences are required as inputs. However, the binding sites collected vary in length. To calculate the data dispersion of binding site length, we used the boxplot statistical method to determine the threshold of binding site length in the dataset and removed a few "abnormal" binding sites in the dataset. According to statistics (Fig. 9) and previous research work, we set the different threshold lengths for each dataset in this study, and sequences of diverse threshold lengths were adopted as input to the model. Binding sites shorter than the threshold length were extended to the threshold length by centring at the point of each binding site, and the upstream and downstream sites were expanded by half of the threshold length each. To avoid noise, the excess sequences were padded with ‘N’ rather than a spliced circRNA sequence. For binding sites longer than the threshold length, because of the small proportion, we temporarily regarded it as the abnormal point and discarded it. Finally, we constructed seven binding site datasets on circRNAs according to the seven RBPs listed in Table 3.

**Table 3:** Seven RBPs involved in human disease by interacting with circRNAs

Datasets	Positive	Negative	Literature			
			RBPs	circRNAs	Disease Name	PMID
DS_AGO2	111,783	111,783	AGO2	hsa_circ_0001346	Lung adenocarcinoma	29704631
				hsa_circ_0001946	Non-small cell lung cancer	31249811
				hsa_circ_0006101	Osteosarcoma	31103262
				hsa_circ_0006117	Non-small cell lung cancer	31160270
				hsa_circ_0007874	Chronic hepatitis B	31148365
DS_AUF1	2906	2906	AUF1	hsa_circRNA_102439	Breast cancer	29973691
DS_EIF4A3	251,183	251,183	EIF4A3	hsa_circ_0001162	Glioblastoma	30470262
DS_FUS	40,918	40,918	FUS	hsa_circ_0000005	Glioma	30736838
DS_IGF2BP3	54,786	54,786	IGF2BP3	hsa_circ_0006156	Gastric cancer	30963578
DS_MOV10	6,003	6,003	MOV10	hsa_circ_0033079	Glioma	30621721
DS_QKI	979	979	QKI	hsa_circ_0007874	Lung adenocarcinoma	30975029

In sequence numerical encoding, each DNA/RNA sequence is represented by a $4^{k}$-dimensional vector called *k-mer* compositional features, in which each feature indicates the normalized frequency of the corresponding *k-mer* appearing in the sequence [45]. This coding method has difficulty capturing sequence order information, especially in detecting motifs. In this study, each binding site sequence is converted to a padded one-hot vector matrix, which is an order-preserving transformation. Specifically, given a sequence $s =^{\prime}s_{1} s_{2} \cdots s_{L} ^{\prime}$, where $L$ is the length of a binding site sequence fragment, $S_{i} \in \left\{ {A, T,C, G,N} \right\}, i = 1,2, \cdots ,L$, which are represented as vectors $\left[ {1,0,0,0} \right],\left[ {0,1,0,0} \right],\left[ {0,0,1,0} \right],\left[ {0,0,0,1} \right],\left[ {0,0,0,0} \right]$, respectively. Here, the padded character ‘N’ is indicated ${ }\left[ {0,0,0,0} \right]$. Our model employs the convolution neural network as the first layer, and padding 0 has no effect on the convolution result. Finally, the binding site sequence is stored as an $L \times 4$ matrix $M$ in the obvious way:

1 $$m_{i,j} = \left\{ {\begin{array}{*{20}l} {1,} \hfill & {if\,\, s_{i} = jth\,base\, in\, \left( {A, T,C,G} \right)} \hfill \\ {0,} \hfill & {if\,\, s_{i} = N\,or\, others} \hfill \\ \end{array} } \right.$$

For example, ${ }if{ }s =^{\prime}NGACAN^{\prime}{ }$, then the representation is shown as follows:

2 $$M = \left[ {\begin{array}{*{20}l} 0 \hfill & 0 \hfill & 0 \hfill & 0 \hfill \\ 0 \hfill & 0 \hfill & 0 \hfill & 1 \hfill \\ 1 \hfill & 0 \hfill & 0 \hfill & 0 \hfill \\ 0 \hfill & 0 \hfill & 1 \hfill & 0 \hfill \\ 1 \hfill & 0 \hfill & 0 \hfill & 0 \hfill \\ 0 \hfill & 0 \hfill & 0 \hfill & 0 \hfill \\ \end{array} } \right]$$

#### Model construction

In recent years, a convolutional neural network (CNN) has been employed to extract the abstract features of genomic sequences; however, the equivariance of these features is not considered in the classification task. In particular, the binding direction of the binding sites is equivalent to that of circRNAs. The capsule network solves this problem by replacing the neurons with capsules, and its output is a vector [44, 46]. The norm of the vector indicates whether a certain type of pattern exists, and the content of the vector represents the equivariance of the features. In this study, we attempted to identify the sequence specificities of circRNA-binding proteins by employing a slight variant of the capsule network. A schematic diagram of our model is shown in Fig. 1. The original features of the sequence are extracted by the convolution operation, and the max-pooling layer is added to downsample the convolution features in our model. Then, the equivariance of the convolution features is obtained by two dynamic routing algorithms. Finally, the norm of the two output vectors indicates the confidence that the sequence is a binding site. Different from the typical capsule network, we removed the reconstruction network and added the max-pooling layer.

Specifically, for a circRNA bound sequence that has been coded as a $L \times 4$ matrix *M*, a convolution operation is used to extract abstract features from matrix *M*. A new abstract convolution feature $conf_{i}$ can be obtained as follows:

3 $$conf_{i} = f\left( {\mathop \sum \limits_{j = 1}^{h} w_{j} *x_{j} + b} \right)$$

where $f$ is a nonlinear activation function ReLU. $x_{j}$ is the *j-th* nucleotide coding, and $w_{j}$ is the corresponding weight. $h$ is the size of the convolution filter, and $b$ is the bias term. Then, a feature map $\left[ {conf_{1} ,conf_{2} , \cdots ,conf_{L - h + 1} } \right]$ is obtained by employing the convolution operation. To downsample the convolution features and acquire the maximum response on each feature map, the final convolution feature is obtained using a max-pooling operation.

To extract the equivariance of the convolution features, the convolution outputs are fed to the primary capsule layer. The function of the primary capsule layer is to convert convolution features into capsule vectors; in our case, the dimension of capsule vector $v$ is set to 8, as in the original capsule network [41]. Because the norm of a capsule vector indicates the probability that the entity presented [41], a new nonlinear activation function is needed for the capsule vector $v$. The norm of vector $v$ is squashed to between 0 and 1 by a squashing function [41] in each capsule. The squashing function does not change the direction of the vector but only changes the magnitude of the vector. The larger the vector is, the closer it is to 1, and the smaller the vector is, the closer it is to 0. The squashing function is shown as follows:

4 $$v_{out} = \frac{{\left\| {v^{2} } \right\|}}{{1 + \left\| {v^{2} } \right\|}}\frac{v}{\left\| v \right\|}$$

$v_{out}$ is the output of the primary capsule layer. Suppose that there are $n$ capsules in the primary capsule layer, and the outputs of the primary capsule layer are $v_{out}^{i} \in \left( {v_{out}^{1} , v_{out}^{2} , \cdots ,v_{out}^{n} } \right)$ as the input vectors for the next layer. Then, the affine transformation of the output vector $v_{out}^{i}$ in the previous layer is performed as follows:

5 $$u^{i} = w^{i} *v_{out}^{i}$$

where $w^{i}$ is the weight matrix. Afterwards, the $T$ times dynamic routing algorithm is applied to $u^{i} i \in \left( {1,2, \cdots n} \right)$ in the digital capsule layer. $T$ is a hyper-parameter. In this study, we set $T$ to be 2. Details of the dynamic routing algorithm are shown in Table 4. $c_{t}^{i}$ is the coupling coefficient that is determined by the dynamic routing process in the algorithm, and $Squash$(*) is the squashing function in Formula 4. Finally, the norm of the vector $v^{t}$ indicates the confidence that the sequence is a binding site or not. Because the prediction of binding sites is a binary classification problem, two 16-dimensional capsules are constructed in the digital capsule layer to represent two states of the input sequences: positive and negative, which represent whether the input is a binding site or not.

**Table 4:** Dynamic routing algorithm

**Dynamic routing algorithm**
**Input**: $u^{i}$ is the output of the affine transformation
**Output**: $v^{t}$ is the output of the $t$ times dynamic routing
**Initialize**: $$b_{0}^{i} = 0, i \in \left( {1,2, \cdots n} \right)$$; T = 2
1: for t = 1 to T
$${2:}\quad c_{t}^{i} = {\text{softmax}}(b_{0}^{i} )$$
$${3:}\quad a^{t} = \mathop \sum \limits_{i = 1}^{n} c_{t}^{i} \cdot u^{i}$$
$${4:}\quad v^{t} = Squash\left( {a^{t} } \right)$$
$${5:}\quad b_{t}^{i} = b_{t - 1}^{i} + v^{t} \cdot u^{i}$$

Except for the coupling coefficient updated by routing, all other parameters in the network need to be updated according to the loss function. We also adopt the Marginloss [41] function in the training stage.

6 $$Loss = T_{c} {\text{max}}\left( {0, m^{ + } - \left\| {v^{t} } \right\|} \right)^{2} + \lambda \left( {1 - T_{c} } \right)\max \left( {0,\left\| {v^{t} } \right\| - m^{ - } } \right)^{2}$$

where $c$ is category, $T_{c}$  = 1 if category $c$ is present, $m^{ + }$  = 0.9, $m^{ - }$  = 0.1 and $\lambda$  = 0.5. The total loss is the sum of the losses of all categories.

#### Motif discovery

As described in a previous study [39], the convolution layers are akin to motif detectors. For each motif detector $M_{k}$, we only consider some position $i$ if $conf_{i} > 0$ in sequence fragment $s$. The position $j = argmax\left( {conf_{i} } \right)$ is selected as a possible motif site, and the subsequence $s_{j \ldots j + h - 1}$ is, extracted, where $h$ is the size of the motif detector. We extract all subsequences by feeding all positive sequences from the test set, and these subsequences are stacked to compute a PFM (position frequency matrix). If the subsequences have a set of special characters ‘*N*’ in the same position, these special characters are aborted, which does not contribute to the PFM counts. Finally, the PFM is transformed into a sequence logo in the standard way.

## Data Availability

The datasets used and/or analysed during the current study are available from the corresponding author on reasonable request. The demo code and data for the model implementation can be found in the GitHub repository (https://github.com/wzf171/circRB).

## Abbreviations

circRNA: Circular RNA
RBP: RNA-binding protein
ROC_AUC: Area under the receiver operating characteristic curve
ReLU: Rectified linear unit
CNN: Convolution neural network
RNN: Recurrent neural network
CRF: Conditional random field
